# The Effect of Carboxymethyl Cellulose Sodium on the Proofing Tolerance and Quality of Frozen Dough Steamed Bread

**DOI:** 10.3390/foods13060870

**Published:** 2024-03-13

**Authors:** Si-Fan Liu, Ke-Xue Zhu, Xiao-Na Guo

**Affiliations:** State Key Laboratory of Food Science and Resources, Jiangnan University, 1800 Lihu Avenue, Wuxi 214122, China; 6210112053@stu.jiangnan.edu.cn (S.-F.L.); kxzhu@jiangnan.edu.cn (K.-X.Z.)

**Keywords:** frozen dough steamed bread, carboxymethyl cellulose sodium, proofing tolerance, proofing degree

## Abstract

This study investigated the effects of dough proofing degree (1.1, 1.3, 1.5, and 1.7 mL/g) and carboxymethyl cellulose sodium (CMC-Na) on the quality of frozen dough steamed bread (FDSB). As the dough proofing degree was increased from 1.1 to 1.7 mL/g, the specific volume of FDSB initially increased and then decreased, with the maximum at 1.3 mL/g, and then dramatically decreased at 1.5 and 1.7 mL/g, accompanied by a harder texture and secession of crust and crumb, which were the detrimental effects brought by over-proofing. The optimal amount of CMC-Na effectively alleviated the deterioration associated with over-proofing, and the proofing tolerance of FDSB was increased from 1.3 mL/g to 1.7 mL/g. Fermentation analysis showed that CMC-Na significantly improved the extensibility and gas-holding capacity of the dough by increasing the maximum height of the dough (H_m_) and the emergence time (T_1_) of H_m_. Frequency sweep tests indicated that CMC-Na improved the plasticity of proofed dough by increasing loss factor tan δ. Significant reductions were found in peak viscosity and complex modulus G* in pasting properties tests and temperature sweep measurements, respectively, suggesting that CMC-Na influenced starch gelatinization and dough stiffening during steaming, which promoted the extension of the network structure, thus facilitating gas expansion and diffusion. These property changes theoretically explained the improvement in the proofing tolerance of FDSB by CMC-Na.

## 1. Introduction

Frozen dough technology originated in the 1970s, mainly due to the need for product flexibility in terms of freshness and as a rapid response to consumer trends [1,2,3]. Nowadays, frozen dough is used in more and more food categories, and Chinese pastry is no exception, as represented by steamed bread [4,5]. Its popularity is attributed to the fact that it follows the trend of food industrialization and has many advantages over traditional techniques [6]. Current buyers are mainly restaurants, canteens, and retail chains due to better flexibility for handling, trading, and retailing, as well as a considerably extended shelf-life [7]. It is also expected that retailers and casual consumers will appreciate and utilize the benefits of frozen dough, which will help to compensate for labor and skill shortages as expertise is concentrated in larger centralized factories equipped with complete frozen dough production lines, which offer greater production efficiency, food security, and quality assurance [8]. Based on manufacturing processes, frozen dough is divided into unfermented frozen dough, pre-/fully fermented frozen dough, partially baked frozen dough, and fully baked frozen products [2,9,10].

Pre-fermented frozen dough is a type of dough that contains yeast and has been fermented for a period of time before quick freezing [9]. As soon as the dough is fermented/proofed to a certain degree, it is quick-frozen immediately [4]. Frozen dough steamed bread (FDSB) is a new application of pre-fermented frozen dough and is obtained by steaming the frozen dough before consumption [11]. In previous studies, the traditional post-processing procedures for pre-fermented frozen dough were thawing, re-fermentation/re-proofing, and steaming/baking, which were performed to obtain more gases and a more porous structure [4,11,12,13,14]. In this study, the frozen dough made for FDSB contained both yeast and a chemical leavening agent, and no thawing or re-proofing was required because the chemical leavening agent produced gas during steaming. Thus, the FDSB in this study was obtained directly from the frozen dough by steaming. This type of FDSB made from pre-fermented frozen dough without pre-cooking, thawing, and re-proofing is characterized by better eating quality, convenience, and food safety, making it popular with restaurants, convenience stores, and supermarkets across China. However, it is challenging to precisely control the proofing degree of dough before quick freezing, which has direct and vital impacts on the gas amount of the frozen dough and finished products [15]. During proofing, a range of physical and chemical reactions take place in the dough that affect the properties and qualities of final products [16,17,18]. Previous studies [13,14,18] reported that the quality of baked bread obtained from frozen dough was much more influenced by the pre-fermentation time than by the freezing rate. Gabric et al. [19] found that the fermentation degree influenced the freezing tolerance of the dough and the volume and texture of the final bread. It was also published that the freezing of over-fermented dough led to bread with a smaller volume than the initial frozen dough [20]. However, these studies were conducted on frozen dough that was designed to be baked, not steamed. Hence, there is a lack of targeted scientific research on frozen dough for FDSB that is ready to steam without thawing or re-proofing.

It is well known that the proofing degree has critical influences on the finished products [21], but it is not currently quantitatively controlled or measured. In practice, the control of the proofing degree has been based on the experience of technicians, which has resulted in less reliable controllability [17]. Over-fermentation/over-proofing is a common production incident that results in the serious degradation of the quality of finished products, such as a lower product volume, firmer texture, and the appearance of collapse and shrinkage. Nevertheless, most researchers have only explored the effect of fermentation/proofing time on the product volume [22], with a lack of exploration of the other qualities and properties, as well as an absence of solutions to overcome the detriments brought by over-fermentation or over-proofing. Based on the background, the impact of dough proofing degree on the property and quality of final FDSB was investigated, and a solution was explored that could alleviate the detrimental effects of over-proofing. The proofing degree affects the quality of final products by changing the gas-holding capacity, viscoelasticity, and extensibility of dough [17,20,22]. Hence, it is a challenge to improve the proofing tolerance of frozen dough and FDSB.

Polysaccharides play an active role in improving the stability and quality of frozen dough. Zhu et al. [23] found that carboxymethyl chitosan significantly enhanced the frozen storage stability and quality of quick-frozen dumpling skins by reducing the degradation of their rheological properties. Rosell, Collar, and Haros [24] reported that hydroxypropylmethylcellulose improved the water absorption, dough developing time, and mixing stability of wheat dough and reduced its weakening during heating. Carboxymethyl cellulose sodium (CMC-Na), also a kind of cellulose derivative [25], might be used to improve the proofing tolerance of frozen dough by changing the dough properties. The objectives of this study were to (a) investigate the quality changes of FDSB made from frozen dough with various proofing degrees, (b) study the effect of CMC-Na on the proofing tolerance of frozen dough and the quality of FDSB, and (c) explore how CMC-Na improved the proofing tolerance of FDSB from the perspectives of dough rheology, pasting properties, and microstructure. This study provides a theoretical basis for the industrial production of FDSB, which is essential to promote the industrialization of Chinese frozen pastry.

## 2. Materials and Methods

### 2.1. Materials

Wheat flour was provided by COFCO Co., Ltd. (Tianjin, China); its protein and wet gluten contents were 10.94% and 31.70%, respectively. The moisture content of the flour was 13.1%. Industrial semi-dry yeast and baking powder were both obtained from Angel Yeast Co., Ltd. (Yichang, Hubei, China), while the sugar came from Zhanyi brand (Yichun, Jiangxi, China). CMC-Na was produced by Shanghai Changguang Enterprise Development Co. (Shanghai, China) with a viscosity of 900 mPa∙s (1% aqueous solution, 25 °C) and a degree of substitution of 0.96 carboxymethyl groups per anhydroglucose unit. Deionized water was used for all experiments and analytical-grade chemicals and reagents were used. 

### 2.2. Preparation of Frozen Dough Steamed Bread 

The amount of other ingredients depended on the amount of wheat flour, and the percentage of wheat flour was calculated as 100%. Other ingredients included 48% deionized water, 1.0% semi-dry yeast, 1.0% baking powder, 3.0% sugar, and different contents of CMC-Na (0.0%, 0.1%, 0.4%, 0.7%, 1.0%, 1.3%, and 1.6%). Yeast was dissolved in deionized water and activated at 25 °C for 3 min. 

In this study, the straight dough method was used and the basic processing procedure for frozen dough consisted of mixing, sheeting and dividing, proofing, and quick freezing. The sugar, baking powder, and CMC-Na were poured into the flour and mixed evenly; then, the water with yeast was added to the flour and mixed in a machine (ARM-2, Thunderbird Co., Surrey, British Columbia, Canada) for 8 min and a uniform predough was made. The predough was sheeted and rolled into a circular strip with a cross-section diameter of 4.5 cm and cut into square-shaped pieces of dough weighing 65 g and with a width of 3.5 cm. Afterwards, the dough was placed in a proofing box (temperature 32 °C and relative humidity 85%); then, the volume and weight of the dough were measured at different time points. The proofing time ranged from 10 to 60 min. It is important to emphasize that the proofing time of the dough was not fixed because the dough with different CMC-Na contents had various proofing speeds (expansion speeds), and the end of proofing was determined by the proofing degree of the dough. The concept of the “proofing specific volume” of dough was proposed in this study to measure the proofing degree of the dough, which was different from the proofing time or final dough volume used in other studies [13,17] and was a more objective indication of the characteristics of the dough itself. The proofing-specific volume was basically the ratio of volume to weight of the proofed dough and ranged from 1.1 to 1.7 mL/g, with higher values indicating a higher proofing degree of the dough. When the indicated proofing degree was reached, the doughs were transferred to a low-temperature refrigerator (DW-40L328, Shjingmi Co., Shanghai, China), quick-frozen for 75 min at −40 °C, and then saved in a sealed package and stored at −18 °C for 7 d in the refrigerator. 

After frozen storage, the frozen dough was ready to steam without thawing or re-proofing. The steamer (SCC102, Rational Co., Bavaria, Germany) was preheated to 96 °C (the highest temperature the steamer could reach) and the frozen doughs were taken out of the refrigerator and unpacked onto trays without thawing or re-proofing; they were then placed directly into the steamer at 96 °C for 20 min. The FDSB was removed from the steamer and cooled at 25 °C for 60 min. 

### 2.3. Specific Volume

According to AACC-approved method 10-16.01 [26], the weight (g) of the proofed dough or steamed bread was measured by an electronic balance, and its corresponding volume (mL) was obtained with a volume tester (VolScan Profiler, SMS Co., London, UK); the volume-to-weight ratio of the steamed bread was the specific volume (mL/g). The proofing-specific volume of the proofed dough before freezing described the proofing degree of the unfrozen and unsteamed dough during the process, which was continually changing in the proofing step, while the specific volume of the steamed bread described the puffy structure after the dough was steamed, which was fixed. Each determination was repeated in triplicate.

### 2.4. Hardness

The steamed bread was cooled at room temperature for 1 h and sliced into 12 mm thick slices; then, the three central slices of each batch of steamed bread were individually measured in a texture analyzer (Model TA-plus, SMS Co., London, UK). The probe used was the cylindrical probe P36 with “return to start” mode selected. The measurement settings were 50.0% strain, 5.0 g trigger force, 1.00 mm/s pre-test speed, 1.00 mm/s test speed, 2.00 mm/s post-test speed, and at least 6 repetitions. The hardness of each sample was recorded as the indicator of texture.

### 2.5. Crust–Crumb Perimeter Ratio

**Figure 1:**
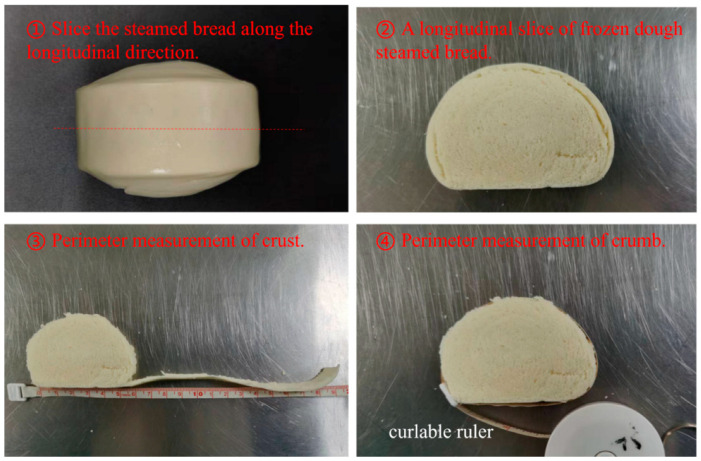
Schematic diagram of crust–crumb perimeter ratio measurement. Measurements were taken in order from ① to ④.

For each FDSB, the three most centered slices were selected, and the perimeter of the outer crust of the slice was measured with a curlable ruler. Then, the crust of each slice was torn off along its natural texture, after which, the perimeter of the crumb was measured, as shown in Figure 1, with a value accurate to the nearest 0.1 cm. The crust–crumb perimeter ratio was calculated as the perimeter of the crust to the perimeter of the crumb. It was used to evaluate the degree of crust–crumb secession of the FDSB. Each determination was repeated in triplicate.

### 2.6. Fermentation Properties

The procedure described by Rezaei, Jayaram, Verstrepen, and Courtin [20] was used, with some modifications; a rheofermentometer (F4, Chopin Co., Villeneuve-la-Garenne, France) was used to evaluate the fermentation properties of the dough with different CMC-Na contents. The specific sampling method was to prepare 300 g of dough according to 2.2; then, the dough was kneaded and pressed to form a flat circle shape and immediately put into the fermentation basket. The dough was squeezed until it fit perfectly with the bottom and sidewall of the tank, 2 kg of weight was placed in the displacement transducer, and the fermentation lasted for 3 h at 32 °C. Related dates were recorded, including the maximum height of the dough (H_m_), the emergence time of H_m_ (T_1_), the time when the dough started to leak CO_2_ (T_x_), the total gas volume produced by the yeast (V_t_), and the percentage of CO_2_ retained in the dough at the end of the test (retention coefficient R). The independent measurements were replicated in triplicate.

### 2.7. Pasting Properties

The dough with different CMC-Na contents was prepared as described in Section 2.2, proofed to a certain degree of 1.5 mL/g (the proofing degree that showed a significant difference for the FDSB), and placed in frozen storage for 7 d. The frozen dough was transferred into a freeze-dryer (Free Zone 2.5, Labconco, Kansas City, KS, USA) and freeze-dried at −40 °C for 3 d to obtain freeze-dried dough powder. According to previous studies [27], freeze-dried powder from dough was used to measure pasting properties by a Rapid Viscosity Analyzer (RVA 4500, Perten Instruments, Sydney, Australia). The freeze-dried powder (3.0 g) and deionized water (25.0 g) were added to an aluminum drum and stirred well to form a suspension with a mass concentration of 10.7 m/m. The suspension was stirred at 960 rpm for 10 s, after which, a constant speed of 160 rpm was maintained. The samples were heated to 50 °C for 1 min, then to 95 °C at a rate of 12 °C/min, held at 95 °C for 2.5 min, returned to 50 °C at a rate of 12 °C/min, and maintained at 50 °C for 2 min. Data were recorded and each measurement was repeated in triplicate.

### 2.8. Dynamic Rheological Properties

#### 2.8.1. Oscillation Frequency

The rheological properties of the frozen dough with different CMC-Na contents were measured according to the modified method reported by Lu [4] and Ishwarya [28] by using a rheometer (Discovery HR 10, TA Instruments, New Castle, USA). In more detail, two groups of dough samples were prepared: one group was made according to the method described in Section 2.2 and the other group was made without yeast. The yeasted dough was tested when its proofing degree reached 1.5 mL/g (the proofing degree that showed a significant difference for the FDSB), the proofing time required for this process was recorded, and the unyeasted dough was measured at the same proofing time. The aim was to investigate the effect of CMC-Na on the rheological properties of the yeasted and unyeasted dough. A diameter of 25 mm was fitted to the rheometer and a small piece of dough (approximately 3.0 g) was placed on the testing bench. The gap width was set at 2.0 mm. After loading the dough samples, excess dough was removed. The dough was balanced for 1 min and coated with silicone oil before measurement. The frequency sweep ranged from 0.1 Hz to 10.0 Hz at a temperature of 25 °C. Prior to the measurements, an amplitude test was performed to determine the linear viscoelastic region so that the strain amplitude was set within a linear range of 0.1% for all samples. G’, G’’, and tan δ were recorded as functions of frequency.

#### 2.8.2. Temperature Sweep

The dough samples used in the temperature sweep were prepared as described in Section 2.2, with a proofing degree of 1.5 mL/g (the proofing degree that showed a significant difference for the FDSB) to avoid differences in the heat transfer rate of dough with various densities and structures, and then thawed at 4 °C for 1 h before testing. Before the formal measurement, an oscillation amplitude test was carried out at various temperatures to determine the linear viscoelastic region and, on this basis, the strain amplitude was set at 0.1%. Following the method reported by Wang [29] and Falade [30], with a few modifications, a diameter of 25 mm was fitted to the rheometer, and a small piece of dough (approximately 3.0 g) was placed on the testing bench. The gap width was set at 3.0 mm. After loading the dough samples, excess dough was removed. The sample was balanced for 1 min and coated with silicone oil before measurement. Samples were oscillated at a frequency of 1.0 Hz. The temperature was ramped up from 4 °C to 110 °C at a heating rate of 3 °C/min, which was different from the real situation inside the steamer, but was a condition that took into account the heat transfer rate of the sample and the temperature difference between the upper and lower plates. The complex modulus (G*) and loss factor (tan δ) were recorded as functions of temperature.

### 2.9. Microstructure

According to previous studies [31], slices of frozen dough steamed bread with a thickness of 12 μm were dyed with rhodamine B and fluorescein isothiocyanate (FITC), followed by observations of the microstructure with 20× magnification and photographing by CLSM (Model LSM 710, ZEISS, Oberkochen, Germany).

### 2.10. Statistical Analysis

All experiments were performed in triplicate and results were presented as mean values and standard deviations. The data were analyzed by SPSS statistics 26 software (IBM, Chicago, IL USA). Statistical analyses were conducted using one-way analysis of variance (ANOVA) with Duncan’s test. The values of *p* < 0.05 were regarded as statistically significant.

## 3. Results and Discussion

### 3.1. Effects of Proofing Degree and CMC-Na on Specific Volume and Appearance of Frozen Dough Steamed Bread

**Figure 2:**
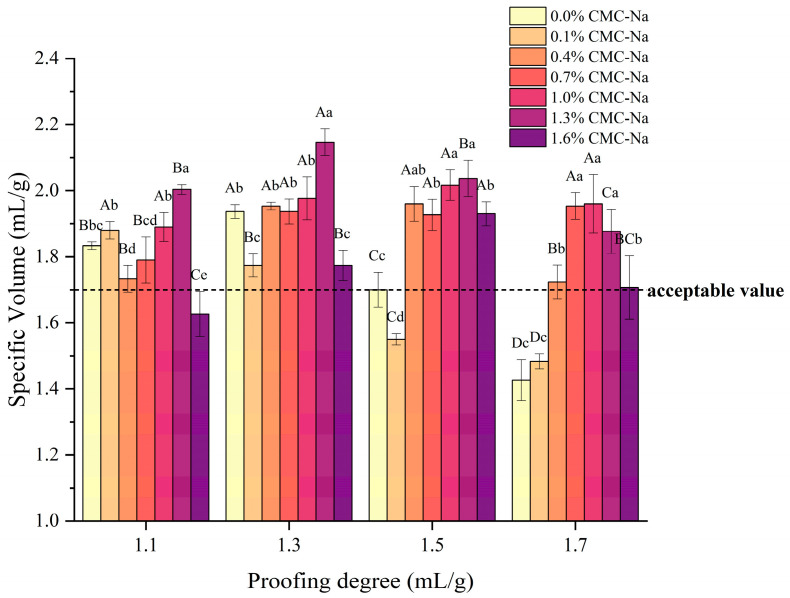
Effects of different proofing degrees and different CMC-Na contents on the specific volume of frozen dough steamed bread (FDSB). Lower case letters indicate significant differences (*p* < 0.05) between the same proofing degree and different contents; upper case letters indicate significant differences (*p* < 0.05) between the same content and different proofing degrees.

**Figure 3:**
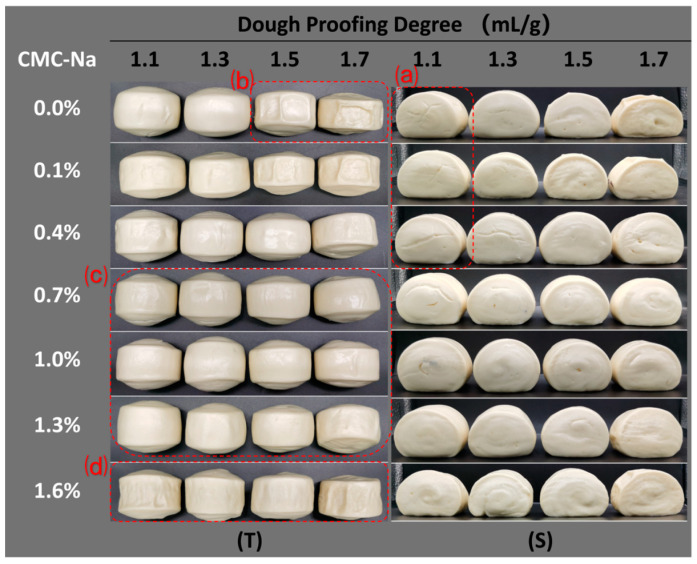
Images of the appearance of frozen dough steamed bread (FDSB) with different proofing degrees and CMC-Na contents, including top view (**T**) and side view (**S**). (**a**) FDSB with side cracks. (**b**) FDSB with serious secession of crust and crumb. (**c**) FDSB of good quality, stability, and proofing tolerance. (**d**) FDSB with shrinkage and collapse.

Specific volume is an important index for evaluating the quality of steamed bread; a higher specific volume indicates that the steamed bread is more fluffy and porous. In this study, the dough went through a proofing process before being frozen, with a certain amount of gas trapped inside, and the proofing-specific volume of the dough was used to represent its proofing degree, which had vital impacts on the specific volume and appearance of the finished FDSB. As shown in Figure 2, the specific volume of the control FDSB first increased and then significantly decreased (*p* < 0.05) as the proofing degree increased from 1.1 to 1.7 mL/g. It can be seen from its trend that 1.3 mL/g was the optimal proofing degree, at which the maximum specific volume of FDSB was obtained. The control FDSB with a proofing degree of 1.1 mL/g had a lower specific volume than the ones at 1.3 mL/g, and side cracks appeared, as shown in Figure 3a part. This result might be related to under-proofing that hindered dough expansion and gas production. The control FDSBs with a proofing degree of 1.5 or 1.7 mL/g had unacceptable specific volumes and shrinkage (Figure 3b part), which might have been associated with over-proofing whereby excessive gas was trapped, exceeding the gas-holding capacity of the dough. Gabric et al. [19] found a similar result in that the volume of frozen bread did not increase with the pre-fermented expansion ratios but, rather, was smaller than the initial frozen dough [14,32]. Thus, for the control FDSB, 1.1 mL/g was under-proofing, while 1.5 and 1.7 mL/g were over-proofing degrees.

During proofing, the gas cells expanded and the gas cell wall became thinner; such changes might have resulted in a decrease in viscoelasticity and a greater susceptibility to the ice crystal damage of the dough so that both the gas-holding capacity and the stretch resistance of the over-proofed frozen dough were weakened [17]. However, 0.7–1.3% CMC-Na significantly improved (*p* < 0.05) the specific volume of the FDSB, especially at over-proofing degrees (Figure 2). Moreover, its appearance had no collapses or shrinkage (Figure 3c part) and so was better than that of the control. These results indicated that the performance of the FDSB with 0.7–1.3% CMC-Na was not drastically influenced by the changes in the proofing degree. Therefore, as evaluated from the aspect of the specific volume and appearance of the FDSB, a certain amount of CMC-Na (0.7–1.3%) successfully improved the proofing tolerance.

The improvements in the FDSB’s specific volume might have been related to the fact that CMC-Na changed the rheological properties of the dough, resulting in the enhancement of viscoelasticity, gas-holding capacity, and extensibility. However, its enhancing effect did not increase with the content. The specific volume of FDSB with 1.6% CMC-Na was significantly lower than the control at the proofing degrees of 1.1 and 1.3 mL/g. The appearance of samples where shrinkage and collapse both appeared (Figure 3d part) also indicated that 1.6% was too much, making the products worse. This might have been due to too much CMC-Na, making the dough too elastic and hindering expansion. Similar results were seen in the steamed bread made with strong flour [33], the reason for which was that dough expansion was hindered by the excessive elasticity caused by the higher gluten protein content.

### 3.2. Effects of Proofing Degree and CMC-Na on Secession of Crust and Crumb for Frozen Dough Steamed Bread

“Secession of crust and crumb” is a common defective phenomenon of steamed bread, whether frozen or fresh, and the severity of secession is also a major criterion for evaluating the quality of steamed bread. In addition, this is a unique phenomenon that distinguishes steamed bread from baked bread and is also different from “oven/steamer-spring” or shrinkage [8,33]. After proofing and freezing, there was a certain amount of gas trapped in the frozen dough, which expanded in the steamer, and the baking powder produced additional gas, resulting in the frozen dough with different proofing degrees having various expansion volumes. In the steaming stage, the dough’s outermost layer firmed earlier under high temperature and humidity, and its viscoelasticity and pore openings were different from the crumb inside [34], so the extra gas could not diffuse out of the system. When the gas amount and releasing speed of the dough exceeded its gas-holding capacity, the connections between the crust and crumb of the FDSB broke, and the broken network was full of accumulated gas. This was due to the imbalance between the gas-producing and gas-holding capacities of the FDSB, eventually resulting in the secession of crust and crumb.

**Figure 4:**
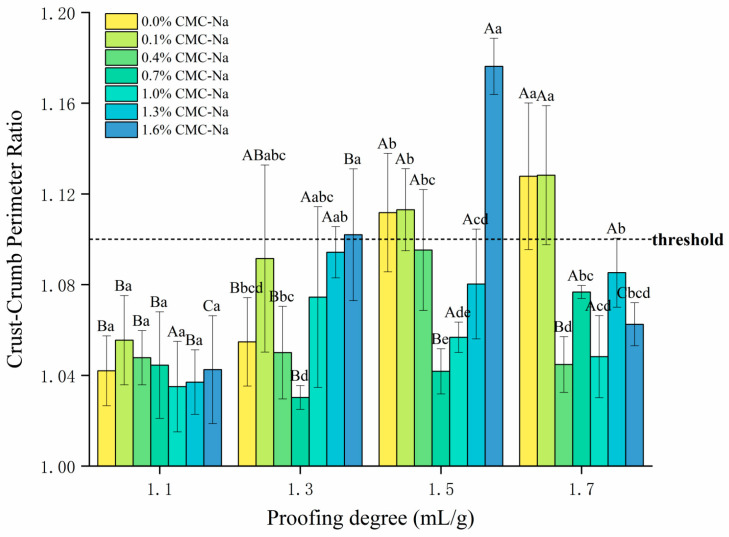
Effects of different proofing degrees and different CMC-Na contents on the crust–crumb perimeter ratio of frozen dough steamed bread (FDSB). Lower case letters indicate significant differences (*p* < 0.05) between the same proofing degree and different contents; upper case letters indicate significant differences (*p* < 0.05) between the same content and different proofing degrees.

**Figure 5:**
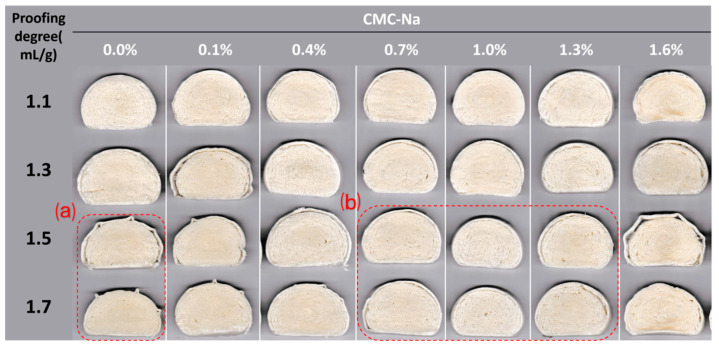
Images of secession of crust and crumb from frozen dough steamed bread (FDSB) with different proofing degrees and CMC-Na contents. (**a**) FDSB slices with serious secession of crust and crumb—the perimeter of the crust was much larger than the perimeter of the crumb and the shapes of slices were irregular. (**b**) FDSB slices without secession of crust and crumb—the perimeters of the crust and crumb were close to each other and the structures of FDSB slices were uniform.

In this study, the perimeter ratio of crust and crumb was used to characterize the secession, and the closer the ratio was to 1.0, the tighter the connection was, and vice versa. Based on the results of pre-experiments, 1.10 was set as the threshold. As shown in Figure 4 and Figure 5, as the proofing degree was increased from 1.1 mL/g to 1.7 mL/g, the crust–crumb perimeter ratio became gradually higher and secession became more severe. When the proofing degree was insufficient (1.1 mL/g) or moderate (1.3 mL/g), the ratio and appearance were both acceptable; once the proofing degree was at or above 1.5 mL/g, obvious secession emerged and the crust–crumb perimeter ratio was more than 1.10 (Figure 5a), suggesting that over-proofing impaired the quality and gas-holding capacity of the FDSB, which was consistent with the deterioration in the specific volume. Long proofing times were one of the reasons for the occurrence of “blisters” in the steamed bread, associated with the weakening of the tensile properties of the dough matrix [17], which were essentially influenced by the gas amount and yeast metabolites. 

The positive effect of CMC-Na was most pronounced in over-proofed FDSB that was proofed to 1.5 or 1.7 mL/g. The crust–crumb perimeter ratio of the FDSB with 0.1% CMC-Na was not very different from the control; however, the FDSB with 0.7%, 1.0%, and 1.3% CMC-Na significantly decreased the ratio at over-proofing degrees, and was still far below the threshold, which can be proved by the comparison in Figure 5a,b. It is worth noting that the positive effect of CMC-Na did not increase with the dosage; on the contrary, a content of 1.6% had a negative impact on the secession of crust and crumb, similar to the influence on the specific volume. A previous study [20] reported that fermentation made dough flow less and break or deform more easily under stress, as well as being less elastic than unfermented dough. CMC-Na might have improved the dough’s extensibility and tensile resistance, which, in turn, alleviated the secession of FDSB and increased the tolerable proofing degree of the frozen dough and FDSB, thus improving their proofing tolerance.

### 3.3. Effects of Proofing Degree and CMC-Na on the Texture of Frozen Dough Steamed Bread

**Figure 6:**
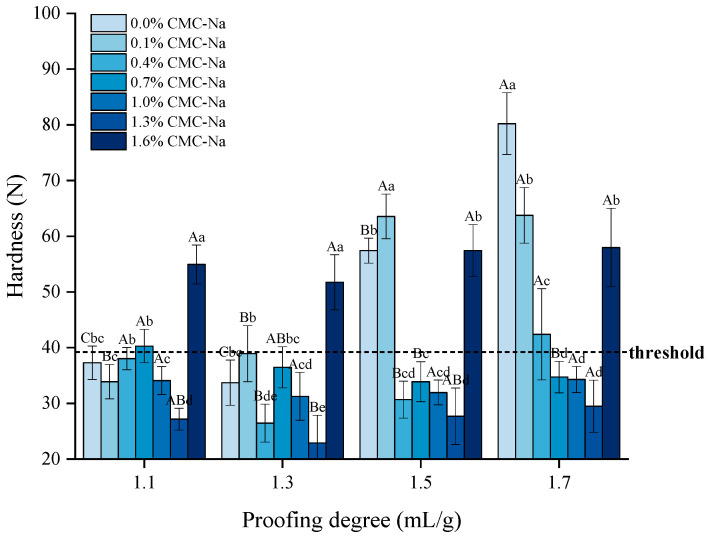
Effects of different proofing degrees and CMC-Na contents on the hardness of frozen dough steamed bread (FDSB). Lower case letters indicate significant differences (*p* < 0.05) between the same proofing degree and different contents; upper case letters indicate significant differences (*p* < 0.05) between the same content and different proofing degrees.

Consumers expect the texture of FDSB to be soft and moderately chewy, which requires its hardness to be within a reasonable range. In the preliminary analysis, consumer sensory evaluation of FDSB with different levels of hardness was conducted, in which the maximum hardness of FDSB acceptable to consumers was 39.2 N (equivalent to 4000 g). Therefore, 39.2 N was determined as the hardness threshold in this study. Figure 6 displays the hardness of FDSB with different proofing degrees and different CMC-Na contents. As the proofing degree was increased from 1.1 to 1.7 mL/g, the hardness of FDSB with 0.0% and 0.1% CMC-Na significantly increased (*p* < 0.05). When the proofing degree rose to 1.5 and 1.7 mL/g, its hardness exceeded the threshold and was not acceptable for consumption. As for the samples with 1.6% CMC-Na, their hardness was too high to be acceptable, regardless of the proofing degree. For the samples containing a certain amount of CMC-Na (0.4–1.3%), the hardness was relatively lower at every proofing degree. Particularly at the over-proofing degrees of 1.5 and 1.7 mL/g, hardness was below the threshold and significantly lower (*p* < 0.05) than the hardness of control samples, which indicated that a certain amount of CMC-Na (0.4–1.3%) resulted in a better texture and palatability of FDSB. This was consistent with the improvement in specific volume (Section 3.1) and can be seen in Figure 5b, where the slices of FDSB with a certain amount of CMC-Na are more porous and spongy. Phenomenally, CMC-Na might act by influencing the diffusion and distribution of gas cells, coupled with enhancing the gas-holding capacity of the starch–gluten matrix, which, in turn, makes frozen dough more tolerant to gas expansion and aggregation during steaming, thus resulting in a fluffier and softer crumb and a higher specific volume of FDSB. 

The effects of proofing degree and CMC-Na content on the qualities of FDSB were analyzed. The comprehensive action of CMC-Na depended on its content and the proofing degree of the dough. The control FDSB showed the best qualities at a 1.3 mL/g proofing degree, while at 1.5 and 1.7 mL/g proofing degrees, there was significant deterioration, including a smaller specific volume, higher hardness, and secession of crust and crumb. These results indicated that for the control FDSB, 1.5 and 1.7 mL/g were over-proofing degrees. However, the FDSB with a certain amount of CMC-Na at the over-proofing degree 1.5 mL/g still maintained acceptable qualities, which suggested that the proofing tolerance was improved, and there were differences in the properties of the frozen dough with different CMC-Na contents at this proofing degree. Therefore, we controlled the proofing degree of all samples at 1.5 mL/g to further investigate the action mechanism of CMC-Na on the proofing tolerance of frozen dough and FDSB.

### 3.4. Effect of CMC-Na on Fermentation Properties of Dough

The fermentation properties of dough can provide essential indicators to infer the characteristics of the dough matrix and yeast, which can determine the proofing performance and quality of final products [35]. Table 1 shows the fermentation parameters of the dough, where H_m_ indicates the maximum height of the dough, T_1_ is the emergence time of H_m_, T_x_ represents the time when the dough started to leak CO_2_, V_t_ is the total gas volume produced by yeast, and the gas retention coefficient R is the percentage of CO_2_ retained in the dough at the end of the test. As shown in Table 1, the addition of CMC-Na significantly increased H_m_ compared to the control sample (*p* < 0.05), and H_m_ was increased by up to 59.9% at a 1.3% content, but there was no significant difference between various contents. Moreover, T_1_ was significantly delayed (*p* < 0.05) in the presence of 0.4–1.6% CMC-Na, and a plateau occurred once the content was increased to 1.0%, at which time, T_1_ was pretty close to 3.0 h. The changes in H_m_ and T_1_ suggested that a certain amount of CMC-Na improved the extensibility and flexibility of the dough and yielded less resistance for the dough to expand during proofing. Consequently, the gas-holding capacity and stability of the dough were improved. This might be related to the fact that CMC-Na is a kind of hydrophilic macromolecule, which dispersed between starch granules and gluten proteins to promote intermolecular entanglements and cross-linking [20], thus enhancing the connections of the matrix and making the dough less prone to deformation or rupture under gas pressure. Combined with the increase in the specific volume and the decrease in the hardness of the FDSB, it can be concluded that CMC-Na had a positive effect on the fermentation properties of the dough, which further improved the quality of the final products. A similar observation was reported by Zhang [15], where the specific volume and springiness of steamed bread were positively correlated with H_m_, while firmness was negatively correlated with H_m_. 

As shown in Table 1, the addition of CMC-Na slightly increased V_t_, but there was no significant difference. Furthermore, the retention coefficient R decreased significantly in the presence of 0.1–0.4% CMC-Na but not with 0.7–1.6% CMC-Na. Analyzing these two changes in combination, it appeared that the gas volume retained in the dough did not increase significantly, which seemed to contradict the change in H_m_. Indeed, this paradoxical result might have been caused by the change in the CO_2_ phase state, which not only existed in gas form but was also absorbed by the aqueous phase in the dough. Based on the fact that the CO_2_ did not lead the dough to expand until the aqueous phase was saturated [36], one hypothesis was proposed that CMC-Na led to a reduction in the aqueous phase of the dough or made CO_2_ less soluble in the aqueous phase. Similar results were observed in the dough with growing sucrose contents, where the H_m_ gradually increased as the retention coefficient R slightly decreased [37].

In addition, the leak time T_x_ advanced slightly but not significantly in the presence of CMC-Na. One possible explanation was that CMC-Na influenced the gas expansion rate of the dough [35], which meant that CO_2_ diffused in the gaseous form earlier, rather than being absorbed into the aqueous phase, thus leading to earlier gas expansion and leakage. Another possible reason for the changes in R and V_t_ was that CMC-Na diluted gluten proteins and competed for the water, which led to a delay in gluten network development [38]. But, at the same time, CMC-Na acted as a filler, with a reinforcement effect on the dough matrix, which facilitated the expansion and maintenance of gas cells, consistent with the results for H_m_ and T_1_.

### 3.5. Dynamic Rheological Properties of Frozen Dough

#### 3.5.1. Oscillation Frequency

The rheological behavior of fermented dough is a complex characteristic influenced by a lot of factors, such as flour quality, dough composition, processing, and yeast metabolites [20,39,40]. Rheological measurements of dough were usually performed without yeast [41] or by degassing the samples before measuring as uneven gas distribution leads to unreproducible results and can also mask tiny changes in the properties of the dough matrix itself [39]. In this study, the gas content and density were the same by controlling the proofing-specific volume of each dough to avoid the property changes in the dough matrix being masked by gas differences. In order to accurately understand the role of CMC-Na in the dough, frequency sweeps were performed on unyeasted dough and yeasted dough.

**Figure 7:**
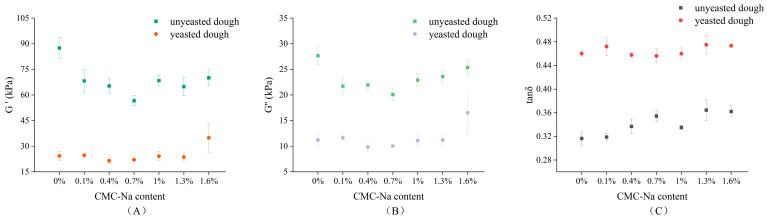
Frequency sweep results of G’ (**A**), G’’ (**B**), and tan δ (**C**) at 1.0 Hz for the unyeasted dough and yeasted dough with different CMC-Na contents.

The improved fermentation performance discussed in Section 3.4 might have been related to a better-balanced viscous–elastic profile of the dough by CMC-Na [38]. The storage modulus G’ (A), loss modulus G’’ (B), and loss factor tan δ (C) at 1.0 Hz of the dough with different CMC-Na contents and various gas-holding states are shown in Figure 7. It was reported that elasticity in the dough was always dominant [42,43], regardless of whether it was holding gas or not. As seen in Figure 7A,B, the addition of CMC-Na decreased the values of G’ and G’’ of unyeasted dough, although no noticeable changes were observed in tan δ, indicating that the elasticity and viscosity were simultaneously weakened and the dough was softened by CMC-Na. This phenomenon was possibly attributed to the molecular configuration and strong hydrophilicity of CMC-Na, which produced smaller and more flexible protein–colloid and colloid–colloid mobile units to influence the development and distribution of larger and more robust protein–protein units; hence, the network strength of unyeasted dough was reduced, which caused a more soft and fluid matrix, resulting in the more free expansion of the dough [31,44,45,46]. Unexpectedly, the softening effects on G’ and G’’ caused by CMC-Na on unyeasted dough were eliminated in the yeasted dough except for the 1.6% content, but tan δ gradually increased with CMC-Na content (Figure 7), indicating that the dominance of elasticity was reduced and the yeasted dough expanded more easily. Combined with the fact that the maximum dough expansion height H_m_ exhibited in the fermentation parameters was significantly increased in the presence of CMC-Na, it could be suggested that the gas retention of the dough was also improved as a higher H_m_ indicated better gas retention [47]. 

As shown in Figure 7, for samples with the same CMC-Na content, both G’ and G’’ of the yeasted dough were significantly lower than those of the unyeasted dough, which might have mainly been due to the gas cells produced by the yeast [39,48,49]. The possible reasons might be that the gas cell expansion led to a stretching impact on the surrounding dough matrix and a breakdown in the gluten network [39,41,46,50], and the polymer interactions in the dough matrix might have been physically hindered by gaseous CO_2_ [50]. From the modulus gap between unyeasted and yeasted doughs, it could be seen that CMC-Na significantly reduced the modulus gap between the two, confirming that CMC-Na effectively improved the resistance of the dough matrix to gas cell expansion and molecular depolymerization, which might be attributed to the electrostatic interactions between CMC-Na and gluten that improved the stability of the gluten network and played the role of “bridge”, which promoted aggregation [45]. The property changes in viscoelastic behavior led to associated changes in final product performance, which was proved by the increased specific volume and other qualities of the FDSB observed above. Therefore, the conclusion that a certain amount of CMC-Na softened the dough matrix and increased its extensibility and stability was drawn. The yeasted dough expanded easily and was more resistant to gas cell expansion; thus, the gas-holding capacity, gas cell stability, and proofing tolerance of the frozen dough were improved, as well as the FDSB quality. 

#### 3.5.2. Temperature Sweep

**Figure 8:**
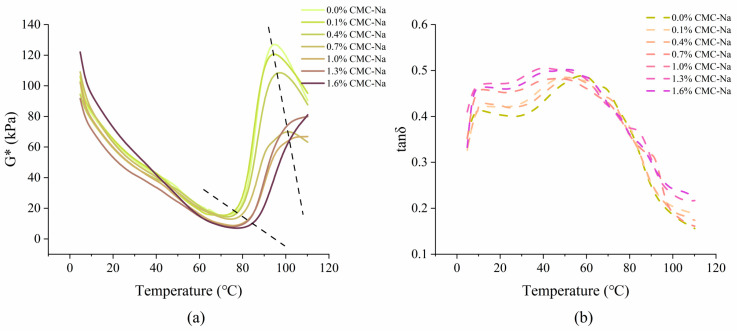
Complex modulus G* (**a**) and loss factor tan δ (**b**) of the frozen dough with different CMC-Na contents during temperature sweep. The dashed lines in sub-figure (**a**) indicate the trends.

The impact of CMC-Na on the complex modulus G* (a) and loss factor tan δ (b) of the frozen dough were studied as functions of temperature, as shown in Figure 8. G* was the composite value of G’ and G’’, which could be used to describe sample strength [27] and might have been correlated with the sample volume and firmness [51,52], and a higher G* indicated a firmer dough. During heating, the beginning of a sharp increase in G* represented the starting point of starch gelatinization [29,53], and the peak G* was related to the maximum intensity of the gelatinization [53]. As shown in Figure 8a, when the CMC-Na content was increased from 0.0% to 1.6%, the temperature when G* began to rise increased from 70.6 °C to 77.8 °C, and the lowest G* was reduced by 52.6%. Moreover, the temperature when G* reached its peak increased from 94.6 °C to 110.2 °C, and the maximum G* modulus decreased by up to 47.4%, and even the frozen dough with 1.3% and 1.6% CMC-Na did not show a turning point of decline when the experimental temperature rose to 110 °C. These observations suggested that CMC-Na delayed starch gelatinization and reduced the strength of the samples, which became apparent as the content increased. This might have been related to the lower water availability for starch granules since CMC-Na had interactions with the water and competed with the starch granules for water. Singh and Bhattacharya [40] pointed out that the rise of G* coincided with the abrupt rise in dough volume. Similar results were also observed by Rouillé [54], who reported that the increase in dough firmness as seen by the modulus increase might be related to the dough–crumb transition since they occurred in the same temperature interval, and the dough–crumb transition thus determined the bread volume and maximum expansion. Thus, the increase and peak of G* might be associated with the time of sample expansion and gas cell opening. It was speculated that CMC-Na not only decreased the dough firmness but also postponed the gas cell rupture in the steamer and increased the gas-holding capacity and extensibility of the frozen dough during its transformation into FDSB, which was the main reason for the improvement in proofing tolerance.

Furthermore, the tan δ also varied along with the CMC-Na content (Figure 8b). In the initial heating stage (4–60 °C), higher tan δ was obtained in the presence of increasing CMC-Na, suggesting that the elasticity of the frozen dough was less prominent and less internal stress was required for the network structure to extend and hold [55], which meant that the gas cell expansion was able to cause a larger volume rise of the FDSB. Consequently, CMC-Na led to a retard of dough stiffening and gas cell rupture, so that the pre-residual gas and new gas from the baking powder both had enough time and space to uniformly distribute in the FDSB, instead of being gathered between the crust and crumb. In addition, the quality of the FDSB discussed above also confirmed that a certain amount of CMC-Na improved the proofing tolerance of the frozen dough by altering its dynamic rheological behavior during both the proofing and steaming processes.

### 3.6. Effect of CMC-Na on Pasting Property of Frozen Dough

As discussed in Section 3.5.2, the functional property of starch dominated in the heating process [52] and had great potential for predicting the quality of products made from frozen dough [9], but premature gelatinization or excessive viscosity leads to earlier gas cell opening and a coarser texture [40]. As a colloid with an excellent water-holding property, the main role of CMC-Na in the dough was to control viscosity [56]. The pasting property parameters of FDSB with different CMC-Na contents are shown in Table 2. Peak viscosity (PV, mPa∙s) was the maximum viscosity reached during the pasting process [57], which comprehensively implies the swelling effect, rupture rate, and water-holding capacity of starch granules. Pasting temperature (PT, °C) indicated the temperature at which viscosity began to increase [58]. CMC-Na had no significant effect on PT except for a 0.1% content, which was not consistent with the results from the rheological measurements because the tests were accomplished with a sufficient amount of water, which was far from a real dough environment. However, PV significantly decreased from 1801.5 mPa∙s to 1449.33 mPa∙s when the CMC-Na content was increased from 0.0% to 1.6%, which might be attributed to the “barrier effect” of CMC-Na [29] as the water absorption and starch granule swelling and leaching were limited, thus providing a favorable environment for gas cell expansion, coupled with increasing the extensibility and gas-holding capacity of the FDSB. Moreover, trough viscosity (TV, mPa∙s) indicated the integrity of starch granules after pasting and their ability to resist shear and temperature [59], which reduced from 1337.5 mPa∙s to 1166.5 mPa∙s with 0.1% CMC-Na and then did not change significantly as the content increased (Table 2), confirming that CMC-Na did hinder the rupture of starch granules to a certain extent. Another parameter breakdown value (BD, mPa∙s) reflected the degree of starch granule destruction and the stability of starch paste [60]. As listed in Table 2, BD dropped down gradually as the CMC-Na content increased, indicating that the starch granules with CMC-Na had greater resistance to hydrothermal disruption during gelatinization [61] and that the fragmentation of starch granules was effectively decreased by CMC-Na. 

The heating and holding stage reflected the property changes of frozen dough during the steaming process, after which, the cooling and final holding stage indicated the property changes of the FDSB. The setback viscosity (SB, mPa∙s) was the gap between the final viscosity (FV, mPa∙s) and TV, reflecting the viscosity increase during cooling, associated with the amount of amylose leached from the starch granules [62]. A lower SB indicated a lower tendency of the starch molecules to rearrange. Once the CMC-Na content rose to 0.7%, both SB and FV significantly decreased with the increase in CMC-Na, suggesting that CMC-Na limited the leaching of the starch granules and interfered with the directional arrangement and recrystallization of the starch molecules so that starch retrogradation was inhibited after steaming. Similar results were observed by Sandhu, Singh, and Malhi [63] in tightly packed and loosely packed starch granules in corn grain. It was hypothesized that CMC-Na enhanced the compact packing of the starch granules in the dough matrix or enabled the development of denser starch clusters, which, in turn, hindered their hydration and swelling. Consequently, combined with the results from the temperature sweep, it can be concluded that CMC-Na acted as a filler and viscosity control agent, which resulted in a more compact matrix of frozen dough with better extensibility and greater resistance to heating and gas expansion [52]. In this way, during the transformation from the frozen dough to FDSB, the network had more space and time to expand because the viscosity of the matrix was decreased, which was beneficial for gas expansion and retention. Hence, CMC-Na improved the proofing tolerance of frozen dough and FDSB by influencing starch gelatinization and gas diffusion. 

### 3.7. Effect of CMC-Na on the Microstructure of Frozen Dough Steamed Bread

**Figure 9:**
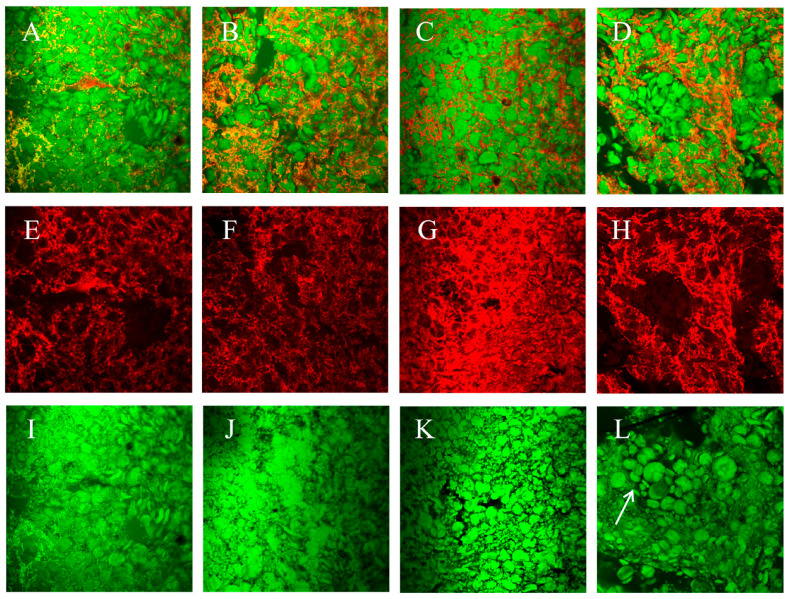
CLSM images of the frozen dough steamed bread (FDSB) with different CMC-Na contents at the 1.5 mL/g proofing degree. Images (**A**–**D**) show the steamed bread matrix stained with rhodamine (**B**) and FITC. Images (**E**–**H**) show the gluten network stained with rhodamine (**B**). Images (**I**) to (**L**) show the starch granules colored by FITC. Letters (**A**,**E**,**I**) represent the CMC-Na content of 0.0%. Letters (**B**,**F**,**J**) represent the CMC-Na content of 0.4%. Letters (**C**,**G**,**K**) represent the CMC-Na content of 1.0%. Letters (**D**,**H**,**L**) represent the CMC-Na content of 1.6%. The arrow in sub-figure (**L**) indicates the “starch clusters”.

Steamed bread is a typical fermented product whose microstructure is described as a soft and porous solid, resembling a sponge structure [52]. The microstructure of steamed bread reflects its mechanical behavior [64]. Apparently, the volume of gas cells increases along with the proofing degree, which couples with migration and aggregation, resulting in the gas cell wall becoming thinner. This research found that the quality of FDSB with different CMC-Na contents appeared to be significantly different when the proofing degree was 1.5 mL/g, and the property changes of the dough at this proofing state have been discussed. To prove that CMC-Na improved the proofing tolerance of frozen dough by postponing starch gelatinization, decreasing pasting viscosity and promoting gas cell expansion, Figure 9 shows the images of FDSB with 0.0%, 0.4%, 1.0%, and 1.6% CMC-Na. From Figure 9A,D, it is evident that the FDSB with 0.0% CMC-Na had the least dense gluten network wrapped around the starch granules. Figure 9G shows that 1.0% CMC-Na caused the greatest cross-linking of the gluten network, proving that CMC-Na improved the connections and flexibility of the dough structure, which might have been due to the enhancement of the intermolecular forces and disulfide bond [17,65] and the improvement of the viscoelasticity of the dough by the interaction of CMC-Na with water. However, a content of 1.6% (Figure 9H) instead resulted in a network breakdown similar to that of the control sample (Figure 9A), which was caused by the physical barrier of starch clusters, as shown by the arrow in Figure 9L. Therefore, the morphology of starch granules was obviously affected. Figure 9I displays starch granules with irregular shapes and ambiguous boundaries, indicating rupture and gelatinization after steaming. As the CMC-Na content increased, more neatly arranged and intact starch granules appeared, and even apparent “agglomeration” was observed, suggesting that starch swelling and rupture were hindered, which was consistent with the results from the RVA and temperature sweep. It was supposed that CMC-Na dispersed among the starch granules and gluten proteins, which acted as a “glue” and “shield” and, with the participation of water, formed a “four-phase system”. Because CMC-Na has a strong water-holding capacity, the interaction of water and CMC-Na might have reduced the water absorption and swelling of the starch granules, which ultimately limited starch gelatinization. Thus, the starch granules in this system were more resistant to vapor and thus less prone to rupture, resulting in a sudden increase in viscosity. In this way, not only the structure stability and extensibility of the frozen dough were enhanced during its transformation into FDSB but also the retention of gas expansion was increased, which ultimately improved the proofing tolerance of the frozen dough and FDSB.

## 4. Conclusions

Currently, the main factor limiting the availability of this kind of FDSB is the uncontrollability and irreversibility of the proofing degree. In this study, it was demonstrated that the proofing degree had a significant effect on the quality of the FDSB, and over-proofing was much more harmful than under-proofing; hence, the concept of “proofing tolerance” was originally proposed to evaluate the quality and processing properties of the frozen dough. The maximum proofing degree that the frozen dough and FDSB could tolerate was effectively improved from 1.3 mL/g to 1.7 mL/g with a certain amount of CMC-Na (0.7–1.3%), and the final products were superior in all aspects. It was concluded that the proofing tolerance was significantly improved by CMC-Na. 

Further discussions about the action mechanism investigated the property changes in the fermentation rheology, dynamic rheology, pasting property, and microstructure of the frozen dough and FDSB. A hypothesis was proposed that CMC-Na dispersed among the gluten proteins and starch granules and, together with water, formed a “four-phase system”, i.e., starch–CMC-Na–gluten matrix, which played a positive role in the proofing and steaming stages, respectively. In the proofing stage, CMC-Na significantly increased the tolerance of the dough to gas cell expansion and improved its gas-holding capacity and extensibility, thus leading to a significant increase in H_m_ and T_1_. During the steaming stage, CMC-Na acted as a starch pasting inhibitor and viscosity controller, which caused the dough matrix to easily expand along with gas cells so that the specific volume and texture of the FDSB were improved. This twofold action theoretically explained the action mechanism of CMC-Na in improving the proofing tolerance of the frozen dough and FDSB. On the one hand, the modified and over-proofed frozen dough had higher gas-holding capacity and extensibility to cope with further gas expansion in the steamer. On the other hand, due to the reduction in network strength and firmness, the dispersion of larger gas cells that were trapped in the dough was easier and more homogeneous, so the frozen dough containing CMC-Na at the over-proofing degree also yielded satisfactory FDSB. Overall, this study provides theoretical and practical guidance for FDSB production and expands the application of CMC-Na.

## Figures and Tables

**Table 1:** The results of fermentation rheology measurements of dough with different CMC-Na contents.

Content of CMC-Na (%)	H_m_ (mm)	T_1_ (h)	V_t_ (mL)	R (%)	T_x_ (h)
0.00	28.80 ± 3.82 ^b^	1.49 ± 0.13 ^c^	1897.50 ± 106.77 ^a^	72.15 ± 1.34 ^a^	0.84 ± 0.02 ^a^
0.10	40.35 ± 0.07 ^a^	1.14 ± 0.01 ^d^	2002.50 ± 31.82 ^a^	66.00 ± 0.71 ^d^	0.73 ± 0.01 ^a^
0.40	44.43 ± 5.17 ^a^	1.83 ± 0.17 ^b^	2015.33 ± 7.64 ^a^	67.27 ± 1.35 ^cd^	0.76 ± 0.10 ^a^
0.70	45.50 ± 2.11 ^a^	2.88 ± 0.10 ^a^	1975.67 ± 46.26 ^a^	70.53 ± 0.93 ^ab^	0.81 ± 0.08 ^a^
1.00	44.93 ± 1.84 ^a^	3.00 ± 0.00 ^a^	1991.67 ± 16.86 ^a^	67.63 ± 0.92 ^bcd^	0.67 ± 0.05 ^a^
1.30	46.05 ± 2.76 ^a^	3.00 ± 0.00 ^a^	1983.50 ± 79.9 ^a^	69.80 ± 0.71 ^abc^	0.68 ± 0.00 ^a^
1.60	44.10 ± 2.79 ^a^	2.99 ± 0.02 ^a^	1962.67 ± 73.76 ^a^	69.47 ± 2.39 ^abc^	0.7 ± 0.11 ^a^

Means in columns with different small superscript letters indicate significant differences at *p* < 0.05. H_m_ indicates the maximum height of the dough, T_1_ is the emergence time of H_m_, T_x_ represents the time when the dough started to leak CO_2_, V_t_ is the total gas volume produced by the yeast, and the meaning of retention coefficient R is the ratio of gas volume that was trapped in the dough at the end.

**Table 2:** The RVA parameters of frozen dough with different CMC-Na contents.

Content of CMC-Na (%)	PV (mPa∙s)	PT (°C)	TV (mPa∙s)	BD (mPa∙s)	SB (mPa∙s)	FV (mPa∙s)
0.00	1801.50 ± 0.71 ^a^	91.63 ± 0.6 ^a^	1337.50 ± 13.44 ^a^	464.00 ± 12.73 ^b^	758.50 ± 6.36 ^a^	2096.00 ± 19.8 ^a^
0.10	1686.50 ± 20.51 ^b^	83.55 ± 9.9 ^b^	1166.50 ± 3.54 ^b^	520.00 ± 24.04 ^a^	791.00 ± 18.38 ^a^	1957.50 ± 21.92 ^b^
0.40	1605.33 ± 39.72 ^c^	91.27 ± 0.03 ^a^	1146.00 ± 35.37 ^b^	459.33 ± 7.09 ^b^	782.67 ± 3.51 ^a^	1928.67 ± 38.03 ^b^
0.70	1527.00 ± 16 ^d^	92.07 ± 0.08 ^a^	1161.33 ± 21.03 ^b^	365.67 ± 18.18 ^c^	689.00 ± 33.45 ^b^	1850.33 ± 23.25 ^c^
1.00	1482.50 ± 23.33 ^de^	92.05 ± 0.07 ^a^	1141.00 ± 24.04 ^b^	341.50 ± 0.71 ^cd^	663.00 ± 7.07 ^bc^	1804.00 ± 16.97 ^cd^
1.30	1457.33 ± 20.5 ^e^	92.07 ± 0.03 ^a^	1134.00 ± 11.53 ^b^	323.33 ± 14.47 ^d^	622.33 ± 46 ^cd^	1756.33 ± 49.33 ^d^
1.60	1449.33 ± 16.5 ^e^	92.08 ± 0.03 ^a^	1164.00 ± 13.75 ^b^	285.33 ± 2.89 ^e^	596.33 ± 22.05 ^d^	1760.33 ± 18.82 ^d^

Means in columns with different small superscript letters indicate significant differences at *p* < 0.05. Parameters included peak viscosity (PV, mPa∙s), trough viscosity (TV, mPa∙s), pasting temperature (PT, °C), breakdown value (BD, mPa∙s), setback viscosity (SB, mPa∙s), and final viscosity (FV, mPa∙s).

## Data Availability

The original contributions presented in the study are included in the article, further inquiries can be directed to the corresponding author.
